# Evolving a Neural Olfactorimotor System in Virtual and Real Olfactory Environments

**DOI:** 10.3389/fneng.2012.00022

**Published:** 2012-10-29

**Authors:** Paul A. Rhodes, Todd O. Anderson

**Affiliations:** ^1^Evolved Machines, Inc.Mountain View, CA, USA

**Keywords:** computational fluid dynamics, odor sensor, olfactory, robotics, simulation, virtual world

## Abstract

To provide a platform to enable the study of simulated olfactory circuitry in context, we have integrated a simulated neural olfactorimotor system with a virtual world which simulates both computational fluid dynamics as well as a robotic agent capable of exploring the simulated plumes. A number of the elements which we developed for this purpose have not, to our knowledge, been previously assembled into an integrated system, including: control of a simulated agent by a neural olfactorimotor system; continuous interaction between the simulated robot and the virtual plume; the inclusion of multiple distinct odorant plumes and background odor; the systematic use of artificial evolution driven by olfactorimotor performance (e.g., time to locate a plume source) to specify parameter values; the incorporation of the realities of an imperfect physical robot using a hybrid model where a physical robot encounters a simulated plume. We close by describing ongoing work toward engineering a high dimensional, reversible, low power electronic olfactory sensor which will allow olfactorimotor neural circuitry evolved in the virtual world to control an autonomous olfactory robot in the physical world. The platform described here is intended to better test theories of olfactory circuit function, as well as provide robust odor source localization in realistic environments.

## Introduction

Brain sensory and control systems evolved to enable action which leads to organism survival. The active interplay between sensation, motor action, and the environment is at the heart of the field of evolutionary robotics (Cliff, 1991; Cliff et al., 1993; Nolfi and Floreano, 2000) and has been studied extensively in the context of visuomotor interaction, so-called “active vision” (Churchland et al., 1994; Floreano et al., 2005). However, evolutionary robotics has received limited attention in the context of olfaction, though olfaction is the more evolutionarily primitive sensory system. While olfactory neural circuitry has been profitably studied as a stand-alone sensory system, combining an olfactory sensory representation with an ability to trigger motor activity has not been attempted. Studying the evolution of sensorimotor transformations is particularly apt in olfaction: olfactorimotor function is reflected in the most evolutionarily primitive chemotactic sensory-motor interactions, such as the spin-and-run behavior observed in paramecium where cilia activity is triggered by chemosensitive ion channels (Greenspan, 2007). Further, the olfactory bulb and piriform cortex dominates the cerebrum in the most evolutionarily primitive vertebrates, hagfish and lamprey (Wicht and Northcutt, 1998), reminding us that olfaction was the foundational cortical sense followed much later by the dedicated visual cortex arising in reptiles (Ulinski, 1990). The physical exploration of a plume has been suggested to be critical in nulling background, making sensory-guided motor control directly related to odorant object identification (Best and Wilson, 2004; Rhodes, submitted). The evolutionarily ancient role of olfaction and the role of exploration in olfactory function suggests that the study of olfactorimotor neural systems will identify principles fundamental to sensorimotor neural systems of all modalities.

A number of intriguing studies have recently pointed to the need for simulations of realistic plume environments in which to develop and test models of olfactorimotor control. Trincavelli (2011) emphasized the joint need for an experimental environment reflecting the turbulence and convection characteristic of natural conditions, but in this work imposed a fixed quasi laminar airflow between source and sensor to enable repeated trials under comparable conditions. In an attempt to develop a database for potential use in studies of olfactorimotor control, Ishida’s group (Wada et al., 2010) obtained maps of convection and sensor readings at a regular grid of points in both indoor and outdoor environments; however, the difficulty entailed in using physical robotic platforms to simply map turbulent plumes was reflected in the fact that minutes separated each of dozens of serial measurements of the ever-changing convective environment, preventing the ability to use the database to reproduce the sensor and convection experience of a robotic agent in traversing any path other than the one used to collect the data. Recently Bennetts et al. (2012) addressed olfactorimotor control in several natural environments with sensors on board three types of physical robot platform, two wheeled and one aerial, equipped with metal oxide (MOX) and photon ionization detector (PID) sensors. They also quantified the effective sampling rate for these sensors (<0.04 Hz for the MOX and <0.15 Hz for the PID) suggesting that reversibility is far slower than the 1–10 Hz relevant to biological sensor responses ranging from canine or rat sniff rates to insect olfactory receptor neurons. Trincavelli and colleagues particularly emphasize the joint necessity of olfactorimotor control experiments with rapidly reversible sensors operating in realistic environments, along with the experimental reproducibility required to systematically test models of olfactorimotor control, whether neurally inspired or not. We have set out to develop a simulation system that meets these goals in this work.

With respect to the control algorithms much work has been motivated by the apparent search strategies adopted by animals, and over the last 15 years a compelling body of work on robotic olfactorimotor control has developed (reviewed in Kowadlo and Russell, 2008; McGill and Taylor, 2011). Most of these studies have however incorporated a more traditional robotic sensorimotor controller, and there has thus far been little work embedding a *neural* olfactory sensorimotor system in a simulated (or real) olfactory environment. In this paper we report on the development of a virtual olfactory plume world with a robotic agent controlled by a neural olfactorimotor simulation. We argue for the use of artificial evolution of the parameters controlling the simulated brain, and then describe how the virtual closed world can be linked to a physical robot, both before and after the development of a physical olfactory sensor with the high dimensionality and rapid reversibility needed to enable plume exploration, so that neural olfactorimotor systems can be evolved in virtual and real worlds in tandem. Describing the components we have chosen to assemble for this integrated system, and addressing some of the constraints encountered and the trade-offs entailed, is the purpose of the present work.

## Materials and Methods

### An environment for the simulation of neural sensorimotor interaction in a virtual plume world

To explore olfactorimotor circuit function we embedded a simulated sensory-motor system in a virtual world which simulates multiple turbulent plume sources and their interaction with a robot agent in real-time. This enables modeling two effects of motor commands on the sensory experience: movement of the sensor position through the plume, and perturbation of the plume dynamics due to agent movement. The virtual plume environment is crucial for developing artificial olfactorimotor machines: robots that can autonomously locate a plume source in the face of their interaction with the plume during exploration. As reviewed in the Discussion below, there are currently no virtual world/robotic simulation packages which integrate real-time computational fluid dynamics (CFD) solvers capable of incorporating flow fields shaped by the features of the environment (convection sources, temperature differences) in which to situate odorant plumes. We first explored available CFD implementations capable of integration into a virtual world, seeking one computationally light enough to update plumes and convection fields interactively during simulation, in order to allow agent position and velocity to affect plume and convection motion. The following is an outline of the set of elements that we suggest need to be assembled to enable the study of neural olfactorimotor interactions:

1. A computationally efficient fluid dynamics simulator integrated into a virtual world.
2. A model of robotic agent, including positions of the olfactory sensors and motor effectors to be connected to neuronal motor representation.
3. A means of communicating the currently sampled odorant concentration from the robot sensor to the brain simulation, and of communicating the motor unit activity levels, or resulting effector control signals, back to the agent.
4. Simulated source odorants, an odorant background, and a sensor array.
5. A simulated neural system including circuitry for sensory representation, motor units, and the linkage between them.
6. A mapping of motor unit activity to the control of effectors on the agent, so that the firing of motor neurons in the circuit simulation moves the agent in the virtual world.
7. A means to select and optimize simulation parameters to guide the construction of a biologically inspired neural implementation of an olfactorimotor system embedded in such a virtual world. We have adopted the large-scale use of artificial evolution for this purpose, and outline some of the challenges entailed.
8. A means to bring all three simulations (sensorimotor neural, robotic agent, and plume CFD) into correspondence with a physical robot, initially in a hybrid real-virtual environment where the plume simulation generates the sensor signals and the output of the neural simulation drive both a virtual and real robot in tandem.

Moving from simulated olfactorimotor environments into physical agents of course requires the availability of a physical olfactory sensor array. A suitable artificial olfactory sensor must be rapidly reversible with a time constant comparable to that of biological olfactory sensors to extract information about plume spatiotemporal changes during exploration (Wada et al., 2010; Trincavelli, 2011), and high dimensional if it is to represent a wide range of odorants, two fundamental prerequisites not jointly met by existing physical olfactory sensor alternatives. We are engineering such a sensor^1^, an array of functionalized carbon nanotube field effect transistors, and briefly reference this work below.

Below, we report the development of a system incorporating the elements enumerated above:

#### Computational fluid dynamics simulator

Arguably, the most important aspect of a virtual world devoted to olfactory search behavior is the fluid simulator. Typically the robotic system has the goal of discovering the location of an object (often called the “source”) by utilizing a stream of sensor signals triggered by the odorant plume emitted by the source. The CFD simulator determines odorant dispersal from the source by constructing a flow field which takes into account multiple physical effects including air entering and exiting the simulation environment (for example, through an open window, under a door-jam, in a heating vent, or out an air return duct), temperature differentials leading to convective currents, and air displacements due to robot movement (detailed further below). The odor plume, represented as either particles or a continuous value in a voxel or other finite element array, is then transported along this flow field. While the simple diffusion of odor molecules is a factor, real plumes of the scale encountered by most laboratory animals are dominated by turbulent flow.

While simulated robotic olfaction plumes are often pre-computed or simulated without ongoing interaction with the moving robotic agent in order to reduce simulation overhead (Cabrita et al., 2010), simulations of the interaction of the body of a moving agent of several centimeters in size or larger with plumes from compact objects (for example sources of food) suggest the perturbation of the plume by the moving agent is very significant, and cannot be plausibly neglected, even in first-order approximation (Dickman et al., 2009). Further, many creatures utilize a variety of means of active intake to enhance olfactory function, including the familiar sniffing characteristic of many mammals and the antennule motion drawing in a stream of water crucial for crustacean olfaction. Such active sensing has been used to advantage in robotic olfactory function (e.g., Ohashi et al., 2008). Simulating olfactorimotor behavior which accounts for perturbation of the plume by movement of the agent, as well as incorporates active intake, requires interactive CFD simulation with an update time comparable in speed to the time step update of the sensorimotor control system.

In selecting a CFD implementation we were therefore forced to balance accuracy with the need for interactive plume updates to account for the movement of the robotic agent under constant (and unpredictable) sensorimotor control by the simulated neural control system. We tested a variety of CFD solutions that enabled multiple odors and interaction between flow field and moving objects but with sufficient computational efficiency to allow plume dynamics to be updated in a time comparable to the neuronal simulation update time. While accurate, traditional CFD software packages such as OpenFOAM, and Comsol Multiphysics are designed to simulate steady-state equilibrium dynamics, rather than the transient dynamics we seek to capture here, and further do not run at speeds approaching real-time. Software designed for generating computer graphics, such as Blender and Houdini, offer exceptional rendering of 3D plumes, but focus on the generation of high resolution effects suited for offline dedicated computation without ongoing interaction with a separately simulated robotic controller. We thus found these software packages unsuited for real-time interactive operation.

While exact implementations of the Navier–Stokes equation system must use very short time steps or implicit solvers in order to avoid instability in the system, Jos Stam has invented a method which employs a simplified version of the Navier–Stokes equations optimized for use in interactive applications such as games (Stam, 1999). To solve fluid equations at video rate, Stam crafts a robust version of the equations which are inherently bounded and thus stable, allowing longer time deltas between simulation steps. Though each time step requires solving a few linear systems and thus is expensive computationally, the fact that each step can represent tens of milliseconds means very few steps are required to run at real-time.

Many implementations of the Stam algorithms exist; for the present work we used Java implementations for ease of prototyping. For rendering a rich graphical interface on a desktop computer we used the MSAfluid fluid dynamics library^2^ within the Processing graphics framework^3^, and for fast computation with no graphics or interface we used an implementation from Karsten Schmidt’s Toxiclibs^4^. The libraries facilitate the creation of a grid of cells, each of which contains a flow force vector and a density variable referred to as “dye.” The regional neighborhood of flow vectors determines the transfer (or advection) of dye from one cell to another, as well as the effect of flow velocity on the flow field. Dye may be injected (or removed) at any cell, and thus is ideal for representing odorant plumes (see Movie S1 in Supplementary Material). This CFD implementation allows in-simulation manipulation of plume-related variables, such the position and intensity of the simulated plume and wind sources. Crucially, this system enables interaction between robotic agent and plume environment that characterizes the genuinely interactive system we wish to study (see Movie S2 in Supplementary Material). Our Processing source code implementing the Stam algorithm in two dimensions is linked here: www.stanford.edu/~tanders/CFD

**Figure 1:**
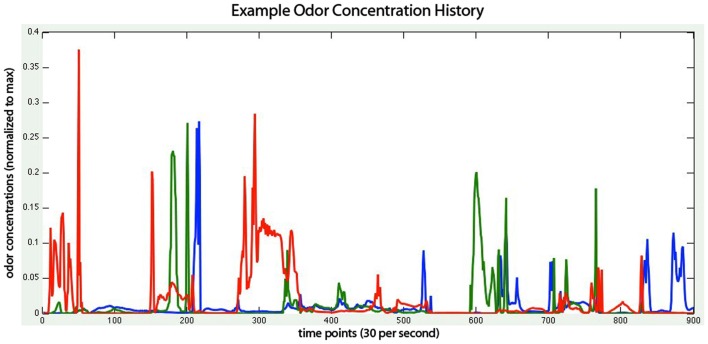
**Concentration of three odors (red, green, and blue lines) experienced by the virtual robot as it moves in the virtual environment, with each point representing 1/30 of a second**. Thus the *x*-axis reflects 30 s of sensor response. Odor concentration is normalized to the maximum concentration found at the odor source. Note the high rate of change of odorant concentration at the sensor surface, and the independence of relative concentration from the two stationary (blue and red) odorant sources.

**Figure 2:**
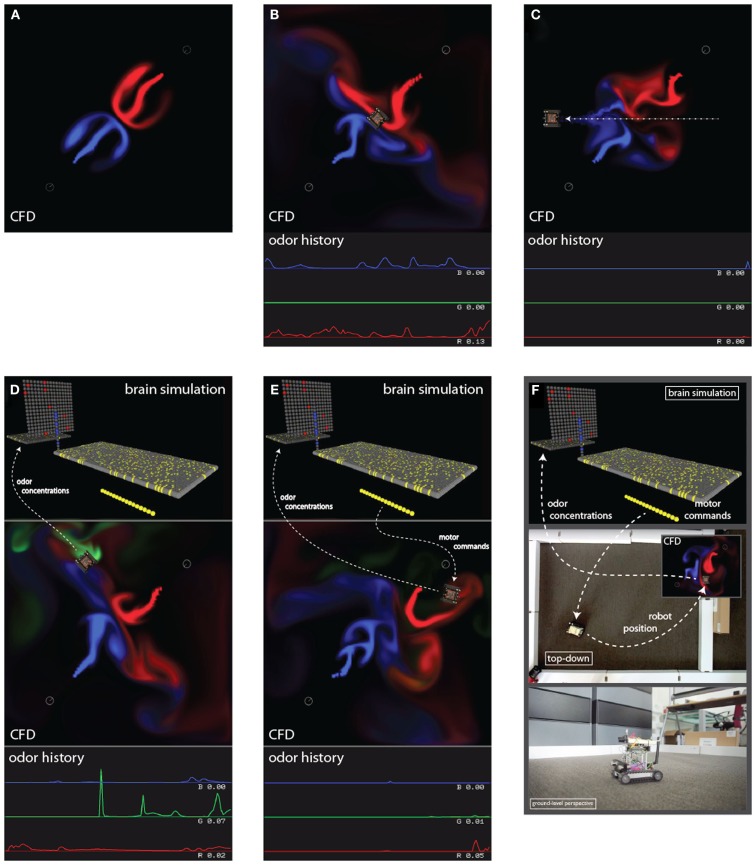
**Series of panels show the incremental build up of our simulation framework**. **(A)** CFD alone, near the start of the simulation. **(B)** CFD with robot positioned in the center, and a plot of the odor concentrations experienced by the robot at bottom; the current reading is on the right, and the plot scrolls to the left. **(C)** Robot perturbing the odor plumes; dotted line is the robot path. **(D)** Simulated brain responding to odor concentrations sent by the virtual robot; dashed line shows information flow. **(E)** Simulated brain sending motor commands to the virtual robot. **(F)** Hybrid real-virtual world, where motor commands are sent to a real robot, which is tracked by video to update the position of the virtual robot in the CFD, and therefore send new odor concentrations to the simulated brain.

**Figure 3:**
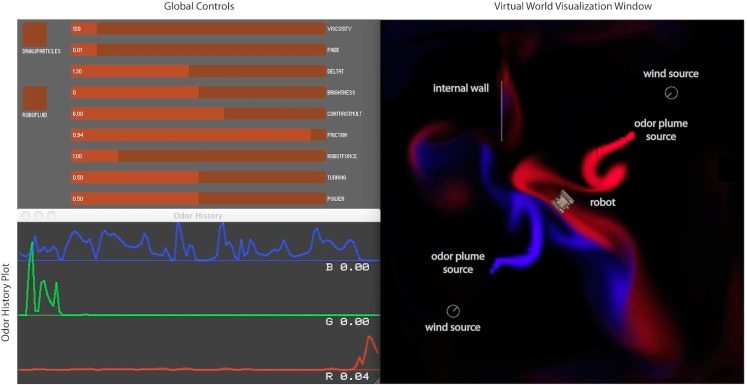
**Virtual World graphical user interface**. The left side shows a panel containing sliders to control simulation parameters, as well as a scrolling plot of odor concentration history experienced by the robot. The right side shows the virtual world rendering, with two plumes (red and blue), a robot (center), two wind sources (gray circles), and an internal boundary.

Currently the virtual world, like the CFD, is two dimensional. Three independent plume sources are modeled, representing three distinct odorants rendered in red, green, and blue, respectively (Figure 1). Odorant concentration is represented by color intensity at the specified x, y cell coordinate within the scalar field (Figures 2A–C). Air movement, modeled as a force vector field, advects the odor plume. A convection source, such as a fan or vent, is incorporated by adding an additional vector to the existing vector in a specified cell or set of cells. The robot body is modeled as a rectangle, with a top-down image of our real robot (a Surveyor SRV-1) mapped on top. The user may reposition and modify the strength of odor and wind sources using a graphical interface. A separate slider-based panel allows adjustment of such global simulation parameters as viscosity, time step delta, odor evaporation, friction between robot and floor, and the magnitude of the robot’s effect on the flow vector field (Figure 3). In addition, a graph of the concentrations of each odor over time is plotted in separate window (see Figure 1, and Movie S3 in Supplementary Material).

#### Robot agent simulation

In order to interact with the simulated plume, we need to represent the robotic agent within that plume. The agent, whether simulated or physical, will include olfactory and other sensors (e.g., for collision detection), as well as effectors to transport the robot (motor-driven tracks) and provide for active air intake mechanisms (“sniffing” fans). We require a virtual world which captures both the physics of moving robots, such as friction, inertia, collisions with obstacles, as well as the fluid dynamics discussed in the previous section. As the robot carries the virtual sensor through the virtual world, encountering different parts of the plume, the odor concentrations at the sensor fluctuate from moment-to-moment in a realistic manner (Figure 1). These concentration values are sent to the brain (see Figures 2D–F), triggering a cascade of activity which eventually activates the motor units. These motor signals are transmitted back to the virtual world to drive the simulated motor effectors used to update the robot position, thus closing the sensorimotor loop via continuous interaction between the virtual environment and separately simulated neural circuitry.

Available virtual worlds designed for simulating robotics are abundant; examples include Microsoft Robotic Simulator (Johns and Taylor, 2008), Webots (Michel, 2004), Player/Stage (Cabrita et al., 2010), and the Robotic Operating System (ROS) promulgated by Willow Garage. Additionally many software packages designed to facilitate creation of video games contain many of the necessary features, including game engines such as Quake (Harvey et al., 2009), Blender (Echeverria et al., 2011), Irrlicht (Ettlin et al., 2005), Unity (Craighead et al., 2007). However, aside from the simplistic particle systems built into many game engines, these existing solutions have not included objects capable of representing odor plumes. One implementation, PlumeSim (Cabrita et al., 2010) built on top of the open source robot simulator Player^5^ may enable integration of plumes into a virtual environment but presently lacks support for interaction between the plume and the exploring robots. Because of the plethora of existing libraries for graphical user interface (GUI) control, fluid simulation, and robot communication, and because of the language used to build the CFD model described in Section “CFD Simulator,” we wrote a simple custom model robot, incorporating friction via velocity-proportional speed decrement, collision proximity detection, in Java.

#### Communication between virtual world and neural simulation

To guide the robot agent toward the source of the plume, we integrated the virtual world with a separately simulated neural sensorimotor system based on the Evolved Machines neural simulator (briefly described below in A Simulated Neural System). The simulated agent in the virtual world was connected to the neural simulation (“brain”) via a two-way socket protocol based on the Microsoft socket library winsock2 and the Processing library called “Network^6^”. Socket connections allow the brain and CFD-virtual world to be modular processes running on separate computers and compatible with any programming language supporting socket communication.

**Figure 4:**
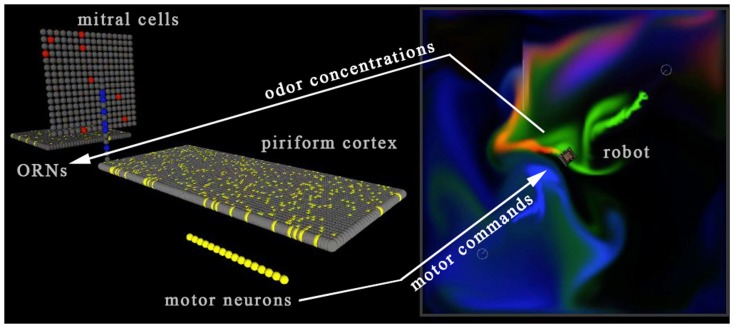
**Screenshot of the neural simulator (left side) reacting to odorant concentration from three odorant plumes (red, green, and blue) at the virtual sensor location (right side)**. A set of 16 motor units activates the forward/backward left/right track treds of the simulated virtual robot, closing the sensorimotor loop with the simulated “brain” receiving sensor information from the virtual world, and via its motor neuron activity sending motor commands into the virtual world. The two otherwise independent software processes were linked by socket-based communications using winsock libraries. See Movie S4 in Supplementary Material.

The amount of data to be passed between the neural simulation and the virtual world is extremely light, just a few tens of bytes per timestep. The concentration of each of the three distinct odorants at the position of the sensor is sent from the virtual world to the Neural Simulator. These values activate the sensor array as described in Section “Simulated Virtual Odorants, Background, and the Sensor Array” below, which in turn activates the mitral and cortical arrays, finally activating the motor units (Figure 4). The net right-left, forward-backward movement signal resulting from the pattern of activity in the motor unit array, encoded as described in Section “A Mapping of the Motor Units to the Control of Effectors on the Agent” below, is then relayed back to the virtual world so that the agent moves through and perturbs the plume environment. Neural activity passes from the sensor to motor units in five simulation timesteps, creating a degree of propagation-time based latency in the neural system.

The robot interacts with the plumes by adding fluid forces to the vector representing the leading edge of the robot chassis. These forces are equal and opposite to the force measured at each CFD cell along this vector, and as the robot moves so do these vectors. This method generates realistic-looking perturbations of the plume during robot movement (see Movie S2 in Supplementary Material).

The robot movement may be controlled in a variety of ways. A human user can steer the robot, using keyboard controls. The robot also has a built-in exploratory behavior, which generates motor commands based on the detection of five sensory states. Two of these states are triggered based on absolute odor concentration being either low or high, and two more on rising or falling concentrations relative to a measurement 1 s prior. The fifth behavioral state is a reversing mode, triggered when the virtual robot collides (reaches zero proximity) with a virtual wall. Finally, the robot can be controlled over the local network, using socket connections. This last method allows communication with human interface devices like joysticks or mobile phones, or more importantly for this project, a remote brain.

#### Simulated virtual odorants, background, and the sensor array

##### Odorants

A set of abstract “motifs” was used to specify both odorants and an array of sensors: odorants were defined by the degree (a real number between 0.0 and 1.0) to which they exhibit each of a set of 10 abstract “motifs” (by analogy to the molecular motifs that correlate with glomerular activation; c.f. Mori et al., 2006). Two odorants could exhibit the same two motifs, though in different relative and absolute degree. The concentration of an odorant is multiplied by motif degree to compute the concentration of the motif present. It is the concentration of motif (not odorant *per se*) that activates the sensors. The background odorant environment comprises a set of additional odorants, constant in space and time but for zero-mean stochastic variation.

##### Sensors

Input to the neural system initiated with the activation of an array of 256 or 1,024 sensors, each defined by their affinity to several of the motifs chosen at random from the 10 available motifs. For the *i*th sensor the affinity associated with motif α is defined as the inverse of the concentration *K_i_*,α of that motif α (not the associated odorant) necessary to elicit 50% maximal sensor activation. With sensors activation level ranging from 0.0 to 1.0, if a given sensor has affinity 1/*K_i_*,α to motif α, and an odorant present at concentration *C* possesses only motif α in degree *d*α, then the concentration of the motif present is *C·d*_α_, and at odorant concentration *C*_50_ ≡ *K_i_,α*/*d*_α_, sensor *i* reaches activation level 0.5. Assuming a first-order interaction between sensor and the concentration of motif α and linear activation of the sensor itself, the steady-state activation level *A*_α_ of sensor *i* in the presence of an odorant at concentration *C* is given by:

$$A_{\alpha }=\frac{C\cdot d_{\alpha }}{C\cdot d_{\alpha }+K_{i,\alpha }}$$

If an odorant exhibits multiple motifs it is assumed that each activates an independent receptor with first-order kinetics which combine linearly and act jointly to activate the sensor; thus in steady-state the activation level of sensor *i* presented with an odorant possessing multiple motifs is:

$$A_{\alpha }=\frac{C}{C+\underset{\alpha }{\sum }K_{i,\alpha }/d_{\alpha }}$$

where the sum runs over all motifs α present in the odorant to which the sensor *i* also has affinity. If one assumes that instead of instantaneous activation receptors have a finite rate of activation and deactivation toward the equilibrium activation level, then we instead have:

$$\frac{dA_{\alpha }}{dt}=r_{f}\cdot \left({Ā}_{\alpha }-A_{\alpha }\right)-r_{\beta }\cdot A_{\alpha }$$

where *r*_α_ and *r*_β_ are the rates of activation and deactivation of the sensor respectively. In this work, it was assumed that sensors activated rapidly in comparison to the rate of odorant variation, and so instantaneous activation of sensors was adopted.

#### A simulated neural system

A very brief description of a subset of the Evolved Machines Neural Simulator used for this work follows. The self-organization of wiring during sensory experience (Rhodes and Taba, 2007), for which this system was built, was de-activated during the present study, which was primarily concerned with putting in place the virtual world and software mechanisms to allow interaction with the a neural sensorimotor circuitry.

##### Piriform cortex

Pyramidal neurons in piriform cortex and Kenyon cells in the insect homolog both receive input on a set of vertically oriented apical dendrites traversed by a horizontal sheaf of afferent axons from the olfactory bulb and antennal lobe respectively. In a typical cortical pyramidal cell these apical dendrites number between 8 and 50, depending upon species, are largely equal in rank, so that given equivalent excitation it is plausible that each branch could make a comparable contribution to somatic depolarization. The Kenyon cells of the Mushroom Body are similar. Inputs from bulb to piriform cortex are very widely distributed (Stettler and Axel, 2009; Nagayama et al., 2010), as is the projection from antennal lobe to Mushroom Body (Jortner et al., 2007), reflecting a remarkable similarity in architecture between insect and vertebrate olfactory systems. Motivated by these anatomical observations, a cortical array of 4,096 neurons (versus 50,000 in the locust Mushroom Body), each with eight identical branches was constructed, with each neuron receiving 160 inputs, 16 excitatory and 4 inhibitory on each branch, from the bulb output units. Thus the piriform cortex incorporated 655,360 synapses. Branches were independent thresholded units (Rhodes, 1999; but see Bathellier et al., 2009), a branch-spike based model of integration encouraged by indirect evidence suggesting that regenerative branch-level spikes are produced in these branches *in vivo* in insects (Laurent et al., 1993), with a neuron activated in turn by the firing of a threshold number of its branches. Branch threshold was a global parameter that homeostatically adjusted during calibration periods. A model neuron of this type can be considered a detector of the presence of a member of a family of subset detectors, well suited mathematically to orthogonalize overlapping inputs that may represent different objects (Rhodes, 2008). As *in vivo* assessment of branch electrogenesis in vertebrate olfactory cortex pyramids has not yet been made, alternative integrative models were considered, including linear dendrites each of which conveyed their unthresholded summed input to the soma, and sigmoidally activated dendrites (Poirazi et al., 2003) which transformed the linear sum of their inputs with a sigmoid and conveyed the resulting value to the soma. Thresholded units performed better in concentration-invariant olfactory classification in preliminary studies and so were adopted for this study.

##### Mitral cells

**Figure 5:**
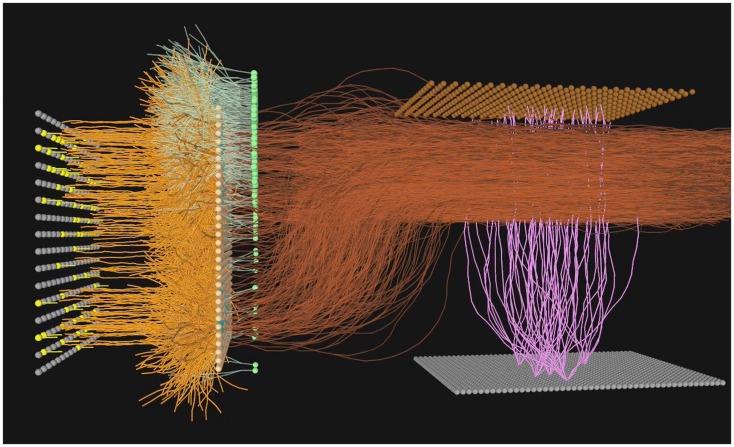
**A simulated neural olfactory system, developed using the Evolved Machines neural simulator**. Here a sensor sheet (far left) innervates a mitral array (orange dendrites). An inhibitory granule cell layer (green cells and dendrites) is synaptically interconnected with mitral cell lateral dendrites via dendrodendritic reciprocal connections. The resulting mitral cell activity is projected (dark orange axons) to the cortex (sheet of gray cells at bottom left; feedforward interneurons shown without dendrites, at top right), where the pattern of activated cortical cells (lavender) represents the sensor information at this timestep. In the first simulations of the sensorimotor system and plume world, the granule cell dendrodendritic interaction was omitted for simplicity, and a set of 16 motor neurons was added, as described in Section “A Mapping of the Motor Units to the Control of Effectors on the Agent” below and illustrated in Figures 2D–F.

A highly simplified “mitral cell” layer consisting of 256 units with a single dendritic branch was utilized, simply to receive the output of a cluster of four of the 1,024 sensor neurons (Figure 5). The interaction between the granule cells and mitral cells present in the vertebrate bulb was neglected in this work, and as a consequence the use of the term “mitral cell” is made only to signify the position of this second layer of units in the flow of activity from sensors to cortex.

##### Synapses and synaptic depression

There were five classes of synapses in this simulated system: (1) sensor neuron to mitral cell; (2) mitral cell to feedforward interneuron; (3) mitral cell to the dendrites of cortical pyramidal cells; (4) feedforward interneuron to the dendrites of cortical pyramidal cells; (5) and cortical pyramidal cell to motor neuron. Each was modeled as an additive weight, with duration of excitatory post-synaptic potential (EPSP) or inhibitory post-synaptic potential (IPSP) an evolvable parameter for each class. Short term synaptic dynamics (depression and facilitation) is present at all cortical synapses (Thomson, 2000), and accordingly here first-order synaptic depression and recover was incorporated at all synapses in the system. During the evolution of system depression and recovery rates for each of the five classes of synapses were among the variables free to evolve to maximize system performance (see below).

##### Local homeostatic adjustment of excitability

It has become increasingly clear that the local homeostatic adjustment of dendritic and neuronal excitability and of synaptic efficacy, via a myriad of co-active mechanisms, is ubiquitous in both invertebrate and vertebrate neural circuitry (Davis, 2006). We have found that when simulating the activity-dependent wiring of developing neural circuits homeostatic adjustment of excitability is indispensable, because the target neurons must have a useful dynamic range early in the wiring process, where there are very few connections per neuron so that excitability must be high to enable some post-synaptic activity, as well as and later when hundreds or thousands of connections have been made, so excitability must decline to present constant activation. Here the thresholds of the individual dendrites of cortical pyramids, as well as firing threshold of the pyramidal cells themselves, independently locally self-adjusted to maintain a target average firing rate. The dendritic and somatic average activity rates were independently evolved, while rates of adjustment and the time constant used to compute the activity average were fixed.

#### A mapping of the motor units to the control of effectors on the agent

While the circuitry and synaptic physiology of the insect and vertebrate olfactory sensory system has been extensively characterized, less information is available, in either insect or vertebrate, regarding the motor system controlled by or affected by olfactory sensory representations. In order to establish an interactive connection between the sensor input from the virtual world to the simulated “brain” and the motor commands sent from the neural system back to move the agent in the virtual world, we connected the cortical array described above to an array of 16 “motor” neurons, each receiving 256 connections from the 4,096 cortical neurons, drawn at random. Given the set of motor effectors on board the simulated agent (forward, backward, left, and right controllers) it was then necessary to define the functional connectivity between the 16 motor units and 4 motor effectors. In other words, when one of these motor neurons fires, what effect occurs at the robot’s motor effectors? We chose a mapping that incorporates the “size principle,” which in physiology refers to the incremental activation of muscle fibers of increasing power to grade effector force in a useful way, allowing for both fine movement, perhaps appropriate for exploration, and more powerful motor action as might be useful during a surge in the direction of a plume upon its location (Henneman et al., 1965). Motor neurons were divided into two populations, left and right, each of which is further divided into another two populations corresponding to forward and backward control. Within each of these four populations a given neuron drives one wheel of the simulated agent (or a track on the physical Surveyor robot) left/right, forward/backward, depending on the population identity of that neuron, with force 1, 2, 4, or 8 assigned to the four motor units in each directional pool.

The motor effector system of the agent we chose was intended to mirror the two treads of a bulldozer-like physical robot, the Surveyor SRV-1, that we used in the hybrid system described below. Thus the four motor neuron pools are all that are needed to represent the positive or negative motor current values transmitted to control the two track motors. The two motors require four graded control populations instead of two, because negative values are not represented by spike rates. To solve the problem of representing negative numbers, which are readily interpreted as negative current values to drive the tread motor backward, the neural system encodes negative values with a second set of units for each tread. The activity of each set of motor units is then added together to compute a net current value for the motor for each tread, which is finally the actual 8-bit integer relayed back to the virtual world to control robot motion, as well as to the physical Surveyor robot in the hybrid system described below.

#### Artificial evolution as a mechanism for refining parameters used in the neural simulation

The sections above outline the components enabling interaction between a simulated robotic agent situated in a virtual olfactory world, with a simulated olfactory sensor activating a neural olfactorimotor simulation, and motor unit activity controlling the actions of the agent. The emphasis has been on achieving an interactive linkage between “brain” activity leading to motor action, and updated sensor stimulation from contact with a plume in the virtual world. The system we describe is clearly highly complex, with a great many parameters associated with the components of the neural mechanisms, including non-linear dendrites, short term synaptic dynamics, and local homeostatic regulation of excitability, all interacting to result in the dynamic relationship between sensor input and motor output. How does one select parameters for this neural system, and make systematically grounded decisions as to which individually well-studied neural mechanisms should be integrated into this system, or excluded in the interest of parsimony and computational efficiency?

We have chosen to approach this problem by defining a performance measure for olfactorimotor behavior of the neurally controlled simulated agent, and then applying large-scale artificial evolution as a means to search the space of parameters as well as neural mechanisms driven by maximization of this fitness. In recent work (Rhodes, submitted) we have evolved simulated olfactory circuitry driven by purely sensory fitness measures, for example the concentration-invariant identification of odorants in stimulus environments incorporating unknown background. In the framework described here, the development of an interactive virtual olfactory world with a robotic agent controlled by the simulated neural olfactorimotor system now brings this work one step closer to true survival-relevant fitness by defining behaviorally defined fitness measures, such as the speed with which the robotic agent achieves a defined proximity to a target odorant source. This measure implicitly combines odorant identification, inherent in reacting to the desired plume amidst other unimportant distracting point sources, and in the midst of distracting background, along with motor control suited to explore and thereby exploit the plume environment to get to the proximity of a source.

##### Evolution operators, and the selection of parameters subject to evolution

An evolution process entails the choice of the ≈50 parameters for one or more initial “parent” parameter sets, the formation of a first generation from these parents, the specification of a subset of parameters to be subject to variation by mutation, the specification of evolution operators (e.g., mutation, recombination, and selection methods) and their parameters. In each trial, then, a particular parameterization of the neural sensorimotor system controls the action and interaction of the robot in the virtual world, defined by a fixed set of odorant sources and convection boundary conditions. For each such trial fitness (e.g., speed of source localization) is computed and stored, and when a full generation of trials is completed the relative fitness of each individual member of the generation is used to select a set of parents for the next generation. Typically a single generation has minimum on the order of 100 distinct parameter sets (“individuals”), with the selection process iterated for order 100 generations, so that in an evolution process order 10,000–50,000 individual interactive neurally controlled robotic runs are performed. The neural system is simulated entirely on NVIDIA GPU hardware employing their CUDA software framework. The speed of this hardware is such that at the scale of order 10,000 neurons, 100,000 compartments, and several million synapses the neural system side of a simulation of order 10,000 timesteps requires a few minutes, excluding virtual world update. If we consider a model of olfactory function, as suggested by Stopfer et al. (2003), that updates cortical representation in a sequence of cycles clocked by the 20–50 ms beta oscillation for vertebrates and invertebrates respectively, and if we allocate four simulation timesteps with which to update the neural system for each such beta cycle (allowing a single timestep to correspond to a 5–10 ms EPSP), then 10,000 simulation timesteps is approximately 2,500 beta cycles, corresponding to approximately 2 min of sensorimotor exploration of the virtual environment. We chose the CFD simulation with the constraint that the time required for these 2,500 updates of 20–50 ms real-time was also order a few minutes of compute time, so that the neural circuit simulation and CFD simulation with which it interacts runs in comparable times. For the 2-dimensional CFD environment with sources and moving robot, at a 140 × 140 grid spacing, simulating a 30 ms update requires approximately 2–5 ms on a modern CPU. Thus the 2,500 such updates in a run the interacting CFD requires approximately 10 s While we do not yet know the update time required for the three-dimensional plume world, including perturbation of the convection field by robot motion, it can be 50-fold longer than the interactive CFD update for the 2-dimensional world and remain comparable in time required for update of a neural system of several million synapses. Therefore, depending on the simultaneity of update a 2 min (real-time) robotic exploration of the virtual plume world controlled by a neural sensorimotor system of the scale noted above is computed in approximately 5 min.

##### The computational resources required, and the use of a farm of GPU’s

Given that a parameter evolution process with a 100-member population evolving for 100 generations requires 10,000 such interactive trials, a single GPU-accelerated compute node would require approximately 1,000 h for a single evolution. If we wish to have these evolutions instead run overnight, say a 12-h period, so each evolution runs results can be analyzed daily, then we can take advantage of the profound parallelism of the evolution process the trials for all members are entirely independent, and so can be run on independent GPU-accelerated nodes, with very data-light communication of fitness results to a control node, and subsequent data-light propagation of the next generation’s parameter sets out to the compute nodes, then 80 such GPU-accelerated compute nodes suffices. At Evolved Machines we are completing installation of a 216-node array of NVIDIA GTX-580-accelerated GPU’s, which we estimate will allow the evolution of an interactive olfactorimotor system, with fitness computed during several minutes of plume exploration in each simulation, over the course of 150 generations computed for a 150-member population in a 12-h period.

#### Hybrid virtual world

This paper describes a method for building a robotic odor source localization system which uses a sensorimotor loop modeled after real neural systems. This brain model contains a large number of parameters, and we would like to tune this model to produce behavior which robustly guides the robot to the source the of odor plume. Two facts have lead us to build a virtual world for simulating the interaction between a robot and a set of odor plumes. The first is that we do not yet have a biologically realistic sensor module which can be mounted on a moving robot. The second is that tuning the brain model requires many simulation runs. As compared to running experiments using real robots and real plumes, simulations vastly increase the number of parameter sets which can be tested in a given amount of time.

However, simulations fail to capture all the complexities inherent in real-world robotics and fluids, for example non-homogeneous ground surface topography and friction, sensor noise, wheels/track slippage, uneven motor power. In order to ensure robust behavior, the simulation-tuned brain models must be exposed to these complexities. A hybrid virtual world, in which certain aspects of the real world are simulated, lets us select which of these complexities our system experiences. In addition, the hybrid world lets us get started tuning the neural model even before we have a reliable mobile olfactory sensor. The ultimate goal is to build a real robot to operate in real environments, and we want to be certain our early experiments are grounded in the complexities of the physical world.

Further, wear and tear would render the robot model non-stationary, and while an effective neural controller of physical robots needs to adjust continually for the drift in the physical model (Bongard et al., 2006), just as animal neural circuits do, that would add a challenge to exclusively using physical robots for this study. It is for this reason that we have developed a virtual plume world, where computing power and a farm of compute nodes running in parallel enables running tens of thousands of plume tracking trials in order 10 h instead of order 1000 h.

For these reasons, we sought to increase the realism of our testing system by putting a real robot in the sensorimotor loop. The robot we used is an SRV-1 from Surveyor Corporation^7^, which is a small tracked robot, about 10 cm × 12 cm in dimension, and 350 g in its most basic configuration^8^. Motor control commands are relayed to the agent over WiFi (802.11 b/g protocol), as are the signals from the variety of sensors that are conventionally mounted on board, which can include a 1.3 M pixel video camera and infrared proximity sensors. To incorporate the robot into the sensorimotor feedback loop, we need to both control the robot motors using signals from the simulated brain, and send odor concentration signals from the robot to the brain. The first task is relatively straightforward; the socket commands consisting of wheel motor control signals are converted from the activity of the motor units as described in Section “A Mapping of the Motor Units to the Control of Effectors on the Agent” above are converted to Transmission Control Protocol (TCP) commands and passed across the network to a server running on the real robot, which then sends a varying amount of current to the left and right motors. The result is that the simulated brain, activated by virtual sensor activity in the virtual world due to contact between an odorant plume and the location of the virtual sensor, result in activation of motor units in the simulated brain which are converted into motor signals which move the physical Surveyor robot on the floor of the lab (see Movie S5 in Supplementary Material).

**Figure 6:**
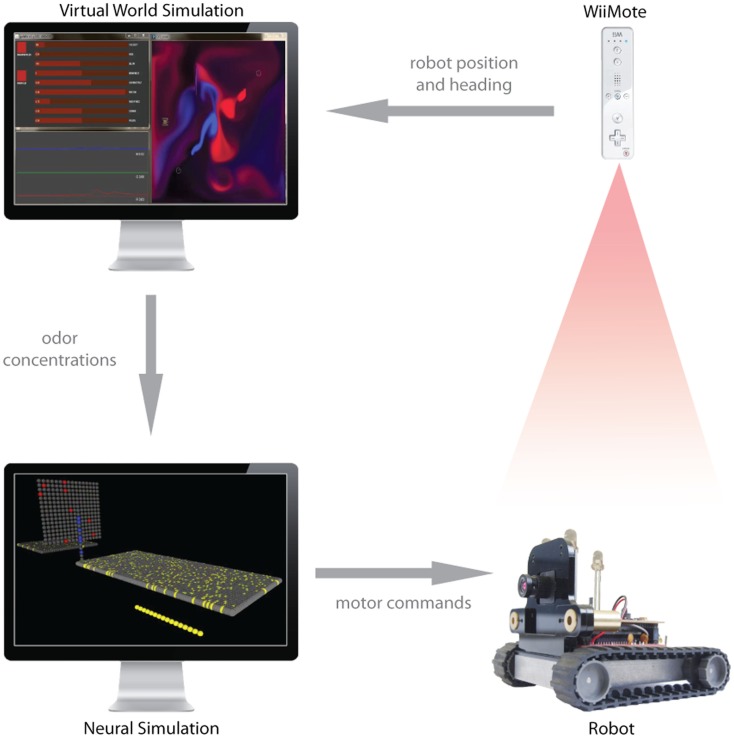
**The hybrid virtual-real world experimental setup**. Upon receiving a motor command, the physical robot (lower right) executes a maneuver beneath the tracking camera (WiiMote, upper right). Based on isolating the coordinates of three LEDs positioned on the robot’s top surface, the coordinates and heading of the robot are sent to the virtual world CFD simulation. The odor concentrations extracted from the robot’s position in the CFD are sent to the computer in the lower left, which is running the brain simulation. The odor concentrations are transduced by olfactory receptor neurons, which spread activation through the neural simulation. When the motor neurons become active, a motor command is sent to the physical robot, which completes the feedback loop.

Since we do not yet have a physical olfactory sensor for the Surveyor robot, we cannot yet have the sensor signals to the simulated brain come from the physical robot. To bridge the gap we have developed a hybrid system, where the new position of the physical robot, controlled by the simulated motor units as just described in this Section and Section “A Mapping of the Motor Units to the Control of Effectors on the Agent” above, is detected and relayed to the virtual world, where it is used to update the position of the virtual robot in the plume world simulation. This requires tracking the real robot in X and Y, as well as determining the heading (for the purposes of sensor localization). To capture robot posture, we use the infrared blob tracking camera on a WiiMote (Nintendo Corporation) to localize three infrared light-emitting diodes (LEDs) we positioned in an isosceles triangle arrangement on top of the robot (Figure 6). The asymmetry of the LEDs lets us determine position and heading from single time points sent over Bluetooth by the WiiMote at 100 Hz, using custom software built with Processing (see text foot note 3) and DarwiinRemote^9^. As the real robot moves, driving by motor commands from the simulated brain, the tracked position and heading are fed into the virtual world to update the posture of the virtual robot. The odor concentrations sent back to the simulated brain are drawn from the robot’s position in the simulated plumes, which are perturbed by the motion of the real robot. Thus the virtual robot is no longer controlled by the neural motor units, but rather mirrors the movement of the physical robot, which is tracked as just described. In this way, we are equipped to begin to study the control of a physical robot by a simulated neural sensorimotor systems activated by interaction between a moving olfactory sensor and a simulated plume, and begin to deal with the attendant irregularities (and non-stationarities) including motor response, floor surface traction, power source variability.

Thus we are able to run 10,000 trials to evolve the parameters of the simulated sensorimotor to optimize as an intermediate step until such time as physical sensors with adequate properties can be engineered and made available, as further described in the next section.

#### The development of a high dimensional, rapidly reversible, compact, low power artificial olfactory sensor using functionalized nanotube FET arrays

In the foregoing we have outlined a system to study the function of olfactory sensory and sensorimotor circuitry with the assistance of a simulated agent embedded in a virtual world, controlled by the simulated neural “brain.” While we expect that the exploration of mechanisms, neural architectures, and parameters choices over order 10,000 trials will continue to require acceleration using a virtual world environments, as noted in the previous section, the olfactory conditions of the real world, and the imperfections of the motor model and effector action (e.g., slippage on the carpet of a wheel) call for transition to a physical machine. To build autonomous olfactory robots driven by reverse-engineered biological neural circuitry requires an olfactory sensor with several crucial properties: (1) High dimensionality. Odorants and odorant mixtures activate biological olfactory sensor arrays identified by expression of a large set of distinct olfactory receptors, ranging from 50 to 150 in the antenna of insects to 900 in the olfactory receptor neuron sheet of olfaction-oriented vertebrates such as canines and rats. The result is a high dimensional dimensional distributed representation, enabling the identification of thousands of objects and environments. (2) Rapid reversibility, so that a time series of odorant measurements can be collected at an adequate rate as an animal explores a plume environment (as emphasized by Bennetts et al., 2012), a dynamic agility which may be central to nulling background and to facilitate odorant identification in complex environments (Best and Wilson, 2004; Rhodes, submitted). The sample rate which has evolved in of highly olfactory animals, around 5 Hz, as a practical target sampling rate. (3) Further to plausibly be mounted on an autonomous physical robot such a sensor would ideally be low power, compact, rugged, and require no consumables. One of us (PAR) is affiliated with a company (see text footnote 1) engineering a sensor comprised of an array of functionalized carbon nanotube field effect transistors which seeks to meet these requirements, and which will be made available to the academic community. With such a sensor, robotic devices could be used in conjunction with the interactive CFD simulations described here to develop and test models of olfactory sensory and sensorimotor circuitry, situating such simulated neural systems in the context for which that circuitry evolved.

## Discussion

### Summary of the present work

In order to study simulations of neural olfactory circuitry in a motor context we have developed an integrated olfactory virtual world incorporating a computationally light CFD simulation of plumes and convection, in which a simulated robotic agent sends odorant concentration values at its location to a separately simulated neural olfactorimotor system. The resulting continuous stream of activation of a simulated sensory array produces activity in the “cortex” of a simulated neural system incorporating approximately 10,000 cells and a million synapses, for now connected to a simple motor array. The units firing in this motor array in turn relay activation signals to a set of motor effectors on the robotic agent, moving it within the virtual world. Its movement there disturbs the plumes and convection fields being simulated in the ongoing computational fluid dynamic simulation, with the resulting update of odorant concentration at the location of the robot sensor being again relayed to the simulated neural system, closing the sensorimotor loop. We optimize neuronal and circuit parameters and refine the choice of neural mechanisms incorporated in this complex interacting system by the systematic use of artificial evolution, driven by fitness measures chosen to reward performance on relevant sensorimotor tasks such a rapidly locating a plume source, amidst other distracting plumes, and unfamiliar background.

### The proposed paradigm in the context of recent work in neural olfactorimotor control

While there is a rich and growing literature exploring olfactorimotor control in both real and simulated environments (reviewed in Kowadlo and Russell, 2008; McGill and Taylor, 2011), the combination of elements listed above, including a turbulent plume simulation perturbed by a robot which is controlled by a realistic neural simulation optimized using artificial evolution, have not yet been integrated. Table 1 presents a summary of the literature identifying the simulated environment, method of parameter optimization, motor performance measure, and algorithm space explored, level of interaction between robot and plume, and other characteristics of each of 12 studies, reported within the last 5 years, so that the neural olfactorimotor simulation environment developed and advocated here can be put in context of the distinguished body of work that has informed this field.

**Table 1:** **A survey of studies of olfactorimotor control presented within the last 5 years**.

Authors and Year	Neural brain	Parameter optimization via artificial evolution	Closed loop	2D/3D	Real or simulated	# odors	Fluid interaction with robot
Rhodes and Anderson (present work)	Yes	Yes	Yes	2D	Both	3	Yes
McGill and Taylor (2011)	No	No	Yes	2D	Both	Up to 3	No
Li et al. (2011)	No	No	Yes	3D	Real	1	Real
Lopez (2011)	Yes	No	No	3D	Real	2	Real
Lu and Luo (2011)	No	No	Yes	2D	Sim	1	No
Cabrita et al. (2010)	No	No	No	3D	Both	1	No
Moraud and Martinez (2010)				2D	Both	1	No
Li et al. (2011)		No		2D	Sim	1	No
Zarzhitsky et al. (2010)	No	No	Yes	2D	Sim	1	No
Ferri et al. (2009)	No	No	Yes	3D	Real	1	Real
Mathews et al. (2009)	Yes	No	Yes	3D	Real	1	Real
Willis (2008)	No	No	Yes	3D	Real	1	Real

*In all but one case, aside from the present study, the sensorimotor control system was not neural, though a variety motor strategies observed in nature were implemented in non-neural control systems. None of the simulation studies incorporated a CFD model that enabled the robot to interact with the plume as it moved, a significant limitation. We advocate the use of a simulated environment to enable the systematic optimization of system parameters, a process that requires the repeated run of plume-robot interaction order 10^3^–10^5^ times, depending on the number of parameters to be jointly optimized*.

With respect to CFD implementation, many studies have implemented turbulent plume simulations using the filament technique of Farrell et al. (2002) or more classic Navier–Stokes (Cabrita et al., 2010). While potentially highly accurate, these equation systems require solution times incompatible with interaction between robot movement (or convection source such as fans, and other mechanisms to achieve sniffing) in real-time during the neural simulation (obviating the ability to run the number of simulations required to do systematic parameter optimization) and so to our knowledge none have enabled the alteration of the plume by a moving robot.

Only a few studies have incorporated neural simulations in guiding olfactory behavior (Mathews et al., 2009; Lopez, 2011), using models built with the IQR simulation framework (Bernardet and Verschure, 2010). Though multiple studies have attempted to emulate the general behavior patterns of odor-seeking insects, often moths (Willis, 2008; Ferri et al., 2009; Cabrita et al., 2010; Lopez, 2011), the use of artificial evolution to systematically search the space of olfactorimotor source localization algorithms (neural or otherwise) is, to our knowledge, unique.

Studies which utilize physical robots (Willis, 2008; Ferri et al., 2009; Mathews et al., 2009; Li et al., 2011; Lopez, 2011) sample real turbulent plume conditions where the perturbation of robot motion is accounted-for, of course, but aside from wind tunnels that ensure laminar flow conditions, it is impossible to ensure that the turbulent plume sensed by the agent is the same from one trial to another. Any method of systematic optimization of the parameters of the simulated system entails a large number of repeated trials which must be sufficiently similarity to make the parameter optimization tractable, suggesting that the use of simulated olfactory environments is indispensable in enabling the exploration of parameter space to an extent that use of physical robots preclude. Once the neural parameters, architecture and mechanisms have been explored in a virtual plume world, incorporation into physical robots is of course a necessary transition to make practical use of the system developed; we therefore emphasized an intermediate step wherein from time to time the physical robot was controlled by the neural motor output, with its resulting position imported to the agent in the plume world, enabling continuous refinement of the virtual world motor model to ensure its relevance to developing motor control of the robot available.

The foregoing review of the literature supports the following enumeration of characteristics of the present system that have not been brought together previously:

1. Control of virtual world agent by a *neural* olfactorimotor system, rather than more conventional robotic control system.
2. Interaction between the simulated robot and the virtual plume and convection field, so that movement of the robot perturbs the plume. This interaction also will enable the addition of (and evolution of the optimum characteristics of) active fluid intake mechanisms such as are employed by biological creatures from lobster to canine to help more efficiently find plume sources.
3. The routine inclusion of multiple distinct odorant plumes and background odor.
4. The systematic and extensive use of artificial evolution driven by olfactorimotor performance (e.g., to minimize the time to locate a plume source) to specify the parameter values for the neural system and to refine the selection of neural mechanisms to incorporate.
5. A hybrid virtual world with the output of the simulated motor units triggering the motion of a physical robot, the resulting position of which updates the location of the agent in the virtual world, as a bridge to incorporate the realities of motor control of an imperfect physical robot. We note that recently Cabrita et al. (2010) presented a hybrid world with a physical agent wherein a Figaro sensor on board moving through a real environment is assessed in parallel with a simulated agent in a virtual world, though the sensory and motor system was not neural, and there was no interaction between the agent and plume.

### Outlook for the future

While we await development of a physical olfactory sensor with the high dimensionality and rapid reversibility necessary to serve as a front end for a real robot, we have developed an intermediate system connecting the purely simulated neural control system and virtual world to the real world. In this hybrid a physical robot, with its motor control imperfections and non-stationarities, is activated by the motor signals from the simulated neural system, with its position on the lab floor monitored and imported to update the position of the simulated agent, which moves through and perturbs the simulated plume. Finally, we describe the development of a new class of high dimensional, rapidly reversible, low power electronic artificial olfactory sensor which, when available, could be the front end for a fully autonomous neural olfactory robotic, with a neural control system co-evolved in virtual and physical environments. When developed, this olfactory sensor platform will be made available to the research community to both explore the development of working olfactory robotic devices and to enable the study of highly neural simulated olfactory circuitry in the sensorimotor context for which it evolved.

## Conflict of Interest Statement

Paul A. Rhodes has an interest in Evolved Machines, Inc. and Nanosense Inc., which are developing simulations of olfactory neural circuitry and their applications in machines, and olfactory sensory for those machines, respectively.

## Supplementary Material

The Supplementary Material for this article can be found online at:http://www.frontiersin.org/Neuroengineering/10.3389/fneng.2012.00022/abstract

## Footnotes

^1^www.nanosensetech.com

^2^http://memo.tv/msafluid_for_processing

^3^http://www.processing.org

^4^http://toxiclibs.org/

^5^http://playerstage.sourceforge.net/

^6^http://processing.org/reference/libraries/net/

^7^http://surveyor.com/

^8^http://www.surveyor.com/SRV_info.html

^9^www.sourceforge.net/projects/darwiin-remote
